# Female Urethroplasty: A Practical Guide Emphasizing Diagnosis and Surgical Treatment of Female Urethral Stricture Disease

**DOI:** 10.1155/2019/6715257

**Published:** 2019-02-18

**Authors:** Marjan Waterloos, Wesley Verla

**Affiliations:** ^1^Department of Urology, Algemeen Ziekenhuis Maria Middelares, Ghent, Belgium; ^2^Ghent University, Faculty of Medicine and Health Sciences, Ghent, Belgium

## Abstract

Female urethral strictures are rare. Guidelines on how to diagnose and treat these strictures are lacking. At present, only expert opinion is available to guide clinical practice. Once the diagnosis is suspected based on obstructive voiding symptoms and uroflowmetry, most clinicians will use in addition video-urodynamics (including urethrography), urethral calibration and cystourethroscopy for confirmation of the diagnosis. Clinical inspection and gynaecological examination are also important. Urethral dilation is usually the first-line treatment despite the lack of long-term success. Female urethroplasty is associated with higher success rates. A multitude of techniques are described but not one technique has shown superiority above another. This narrative review aims to provide a clinical guide for diagnosis and treatment to the urologist motivated to perform female urethroplasty.

## 1. Introduction

Female urethral strictures are rare but can cause severe symptoms impacting the patient's quality of life. About 10% of women with obstructive voiding will have a true (“anatomical”) urethral stricture [1–3]. First-line treatment usually consists of dilation(s) but long-term cure rates are disappointing [4]. In males, several techniques of urethral reconstruction (urethroplasty) have been described and entail extensive experience at high-volume centers with high cure rates [5]. On the contrary, experience with female urethroplasty and the literature about it are scarce with only a few case series with limited follow-up. The rarity of the disease, the lack of experience, and the fear of functional complications (e.g., urinary incontinence) might hamper urologists to perform female urethroplasty. The aim of this narrative review is to provide the urologist treating female urethral strictures a practical guide in which diagnostic modalities are available and to provide a well-illustrated summary of the most commonly used techniques of female urethroplasty.

## 2. Surgical Terminology

**Figure 1:**
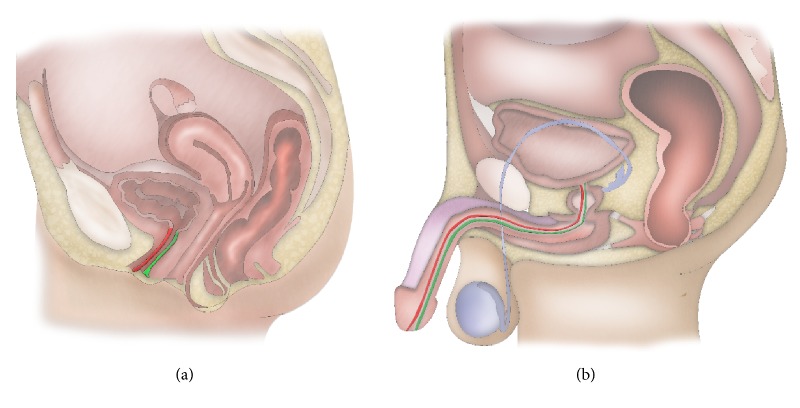
Female versus male urethral anatomy.* (a) = female urethral anatomy; (b) = male urethral anatomy. Green = ventral urethra; red = dorsal urethra.*

The terminology used in female urethroplasty can be confusing and needs further clarification. The definition of dorsal and ventral to describe the location at the urethra is derived from male urethroplasty but is from an anatomical point of view not logic in females [3]. In males, the ventral part of the pendulous urethra is the part pointing forward during erection whereas the ventral part of the bulbar urethra is pointing downwards and even backward at the membranous urethra (Figure 1). In females, the ventral part of the urethra is the part pointing backward, towards vagina. The dorsal part is pointing forward towards the pubic bone. The anterior vaginal wall is the part of the vagina in direct contact with the urethra and bladder whereas the posterior wall is in contact with the rectum (Figure 1). A proximal stricture is a stricture close to the bladder neck, whereas a distal stricture is located close to the urethral meatus.

## 3. Preoperative Evaluation

**Figure 2:**
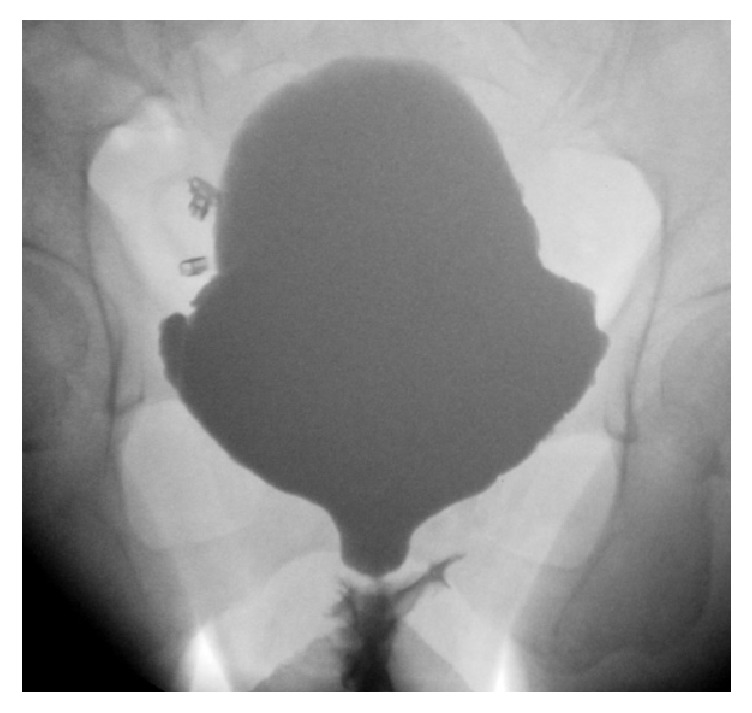
Wine glass image.* Voiding cystourethrography with a distal female urethral stricture and prestenotic dilation.*

A urethral stricture will cause obstructive voiding which is clinically translated into a weak urinary stream, sensation of incomplete voiding, straining, frequency, and nocturia [6, 7]. Many women also experience pain during micturition and urgency [1, 3, 7, 8]. Because of residual urine, these women are at risk for developing recurrent urinary tract infections [3, 6, 7, 9]. In women with lower urinary tract symptoms, uroflowmetry must be part of the diagnostic work-out and a plateau-shaped curve is suggestive for a stricture [10]. A gynaecological examination is indispensable as it might directly reveal a meatal stenosis and the presence of lichen sclerosus, pelvic organ prolapse, or periurethral abnormalities. This examination must also emphasize the quality of local tissues which might be used for urethral reconstruction [1, 2]. The inability to pass a 14 Fr urethral catheter is almost pathognomonic for the presence of a urethral stricture, although there is no strict definition of the normal caliber of the female urethra [3, 4, 11]. Cystourethroscopy might directly visualize the stricture but provides no information about the stricture length. In case of meatal or distal urethral strictures, insertion of the scope might not be possible, especially for the very narrow strictures [4]. Postvoidal ultrasonography can show residual volume inside the bladder [8]. Vaginal ultrasound using an 8 MHz probe can show the presence of the stricture after instillation of gel through the meatus. Retrograde urethrography, the standard evaluation in males, is not practical in females [8, 12]. Instead, antegrade voiding cystourethrography (VCUG) must be used. Filling of the bladder is accomplished by either suprapubic access, if a suprapubic catheter has been placed, or passing a small-caliber (e.g., 5Fr feeding tube) catheter through the stricture inside the bladder. Images are made at start, with full bladder, during voiding and after voiding. Bladder diverticula might be present as well as vesicoureteral reflux. During voiding, the urethra proximal to the stricture will show dilation with abrupt narrowing at the stricture site (Figure 2, “wine glass image”) [6, 13]. Thus, VCUG will provide information about both the location (proximal, mid, and distal) and the length of the stricture.

**Figure 3:**
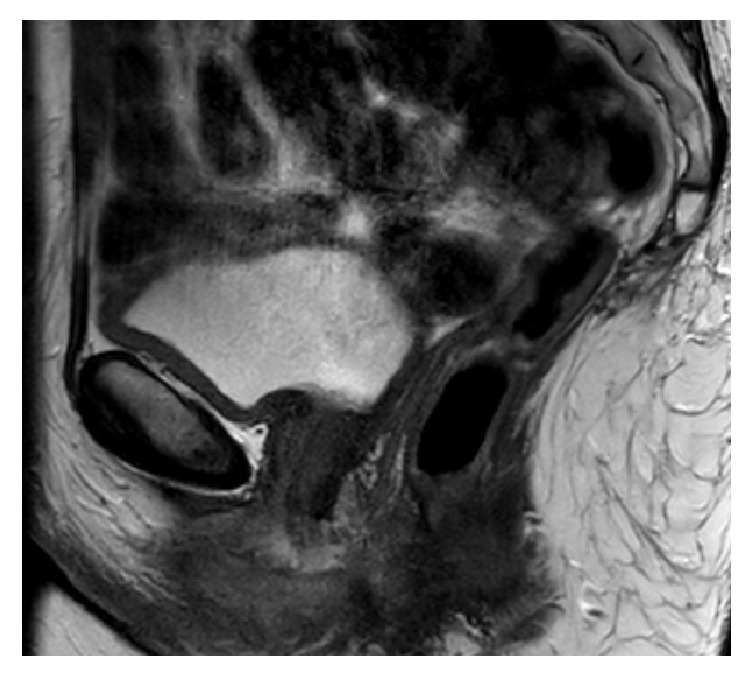
MRI of the female pelvis.* The urethra is clearly visible without the presence of a urethral diverticulum or periurethral abscess.*

Video-urodynamics combines this imaging with pressure-flow studies and provides as such a more complete evaluation [1, 4, 7, 9, 10]. In case of any doubt of concomitant abnormalities (urethral diverticula, abscess formation, etc.), pelvic MRI will provide useful anatomical information [2, 4, 10] (Figure 3).

A few days before operation, a urine culture must be performed and antibiotics must be started in case of infection the day before surgery according to the antibiogram.

No guidelines exist on which diagnostic modalities must be used during the work-out [3], but before start of urethroplasty, the surgeon must have obtained sufficient information on the presence, extent, and location of the stricture as well as on the quality of surrounding structures in order to be prepared for the urethroplasty.

## 4. Surgical Guide

### 4.1. Patient Positioning and Preparation

**Figure 4:**
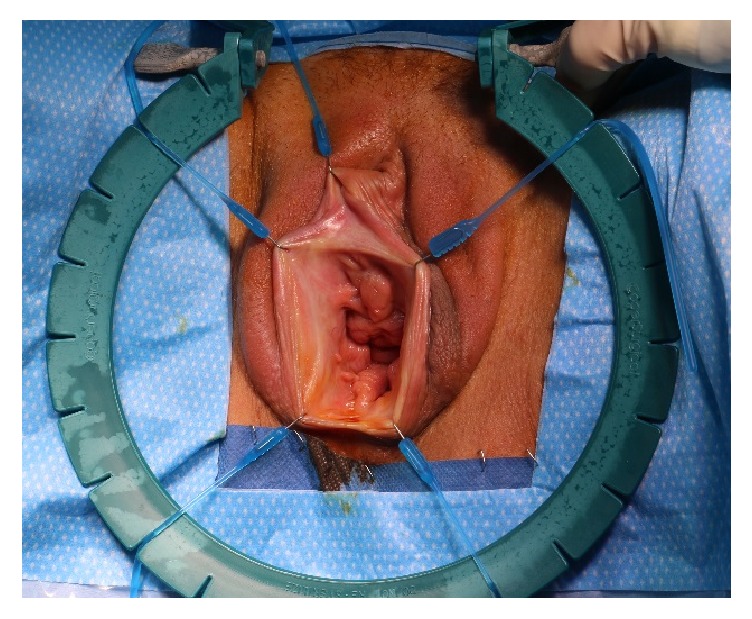
Exposure of the female urethra and vagina using the lone-star retractor.

In postmenopausal women, intravaginal estrogens may be administrated to treat vaginal mucosa atrophy [14]. In order to have a stable and mature stricture, urethroplasty must be postponed 3 months after the last dilation or urethrotomy [15]. All patients are placed in the lithotomy position. If a suprapubic catheter is present, the bladder is instilled with 100cc of 1:1 diluted povidone-iodine solution. The labia minora are retracted by sutures or a lone-star retractor in order to have a good exposure of the vestibulum, the urethral meatus, and the vaginal introitus (Figure 4) [9, 10]. Vaginal access (and access to the more proximal urethra) is facilitated using Doyen's vaginal blade retracting the posterior vaginal wall [16]. A guidewire is placed through the urethra inside the bladder in order to avoid creation of false passage during opening of the stricture [17]. Suture materials for urethral reconstruction are absorbable sutures 4.0 (adults) or 5.0 (children–adolescents).

### 4.2. Surgical Technique

#### 4.2.1. Heineke-Mikulicz Meatoplasty

The stenotic meatus is ventrally incised in a longitudinal fashion until healthy urethral mucosa is reached that allows passage of a 20Fr catheter. The borders of the urethral mucosa are sewed to the borders of the vaginal mucosa in a transverse fashion with separate sutures [1].

#### 4.2.2. Flap Urethroplasty

**Figure 5:**
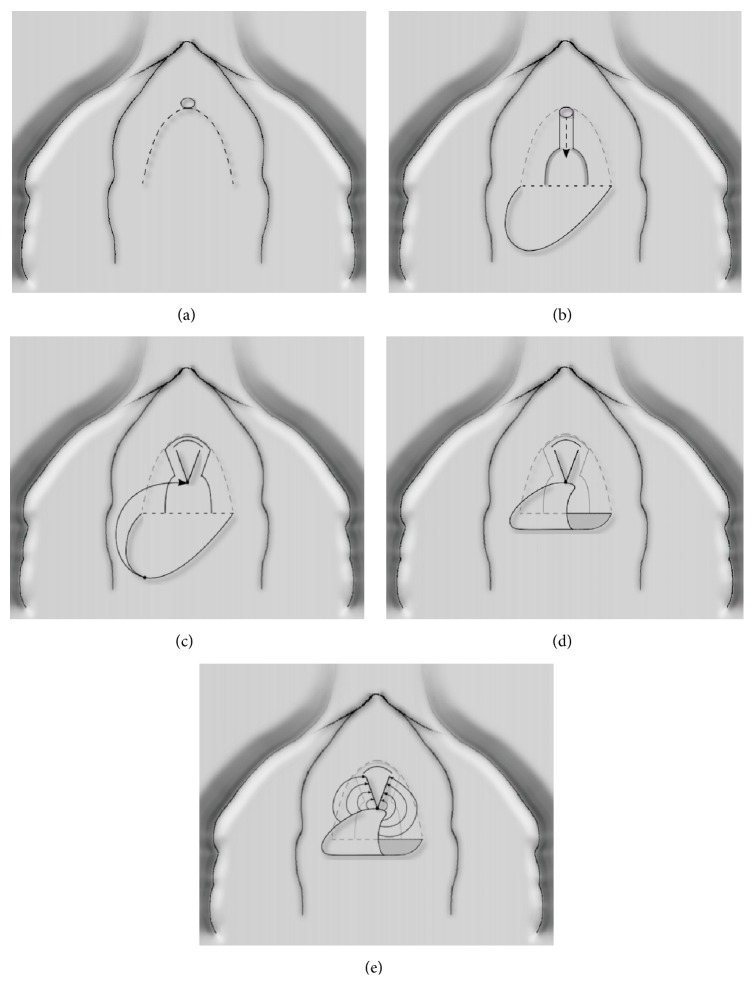
Anterior vaginal wall flap (“Blandy flap”).* (a) = inverted U-incision; (b) = ventral stricturotomy; (c) & (d) = suturing the tip of the U-flap to the proximal part of the opened urethra; (e) = further suturing the edges of the flap to the urethral edges.*

*Anterior Vaginal Wall Flap (“Blandy Flap”) (Figure 5)*. An inverted U-incision is made at the anterior vaginal wall, just below the ventral urethral meatus. The flap is dissected away from the ventral urethra over 3 cm with preservation of the submucosal layer containing the vascular pedicle of this flap. The ventral side of the urethra is incised until healthy proximal urethral mucosa is identified allowing passage of a 20Fr catheter. Stay sutures are placed on both sides of the opened urethra in order to facilitate exposure. The flap is turned towards the opened urethra and the tip of the U-flap is sutured to the proximal part of the opened urethra with 3 sutures. The edges of the flap are further sutured to the edges of the urethra with running or interrupted sutures on both sides until the level of the external meatus. The remaining base of the flap is sutured to the borders of the vaginal mucosa with separate Donati sutures [15].

**Figure 6:**
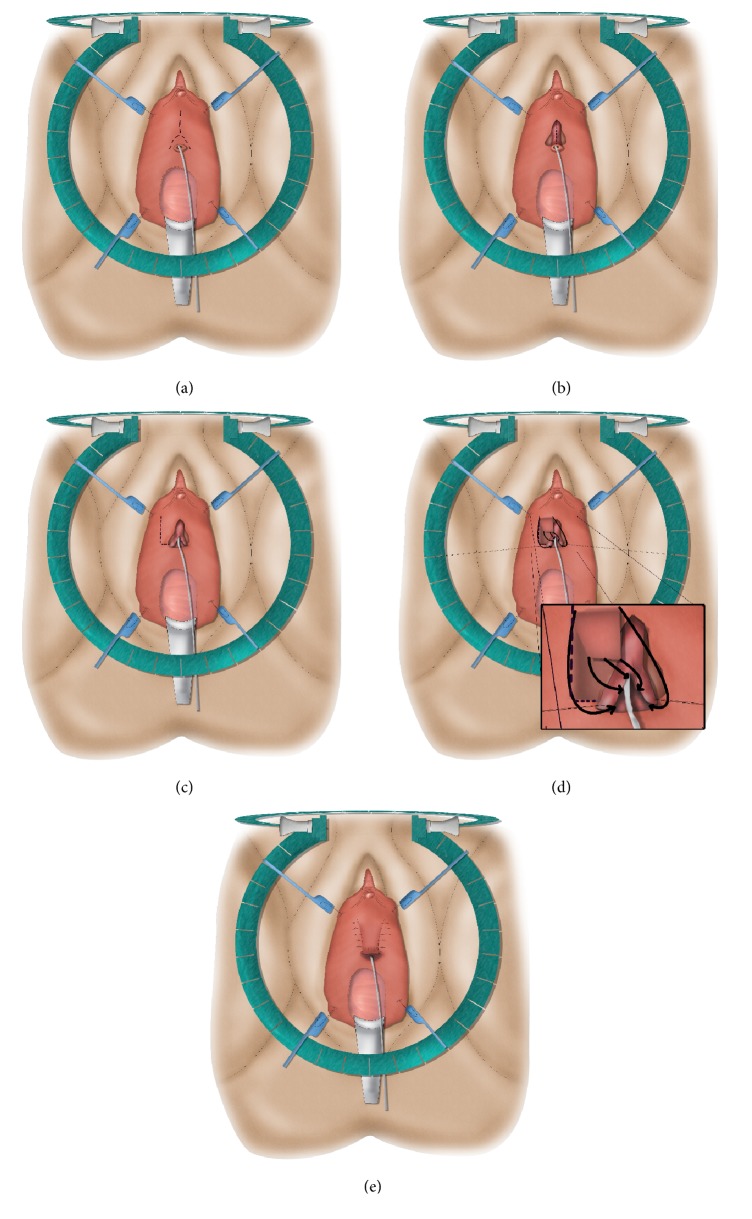
Vestibular flap (“Montorsi flap”).* (a) = inverted-Y incision; (b) = dorsal stricturotomy; (c) = mobilization of the vestibular flap; (d) = suturing the tip of the flap to the proximal end of the opened urethra and the edges of the flap to the urethral edges; (e) = suturing the base of the flap to the vestibular mucosa.*

*Vestibular Flap (“Montorsi Flap”) (Figure 6)*. An inverted-Y incision is made at the dorsal urethral meatus. The distal urethra is dissected away from the clitoris and surrounding suburethral tissues but without accessing the ventral urethrovaginal plane. Once the dorsal urethral wall has been sufficiently exposed, a dorsal urethral incision is made. The strictured urethra is further opened until healthy proximal urethra is encountered allowing passage of a 20 Fr catheter. Stay sutures are placed as described above. According to the length of the stricture, a 1.5-3cm long and 1 cm wide vestibular flap is mobilized from the right or left side, just aside the vertical vestibular incision. A flap with rich blood supply is needed and as a consequence superficial submucosal dissection must be avoided. The tip of the flap is sutured to the proximal end of the opened urethra with 3 separate absorbable sutures. The borders of the flap are further sutured to the borders of the opened urethra on both sides with a running suture. The base of the flap is finally sutured to the vestibular mucosa with interrupted absorbable Donati sutures [7].

**Figure 7:**
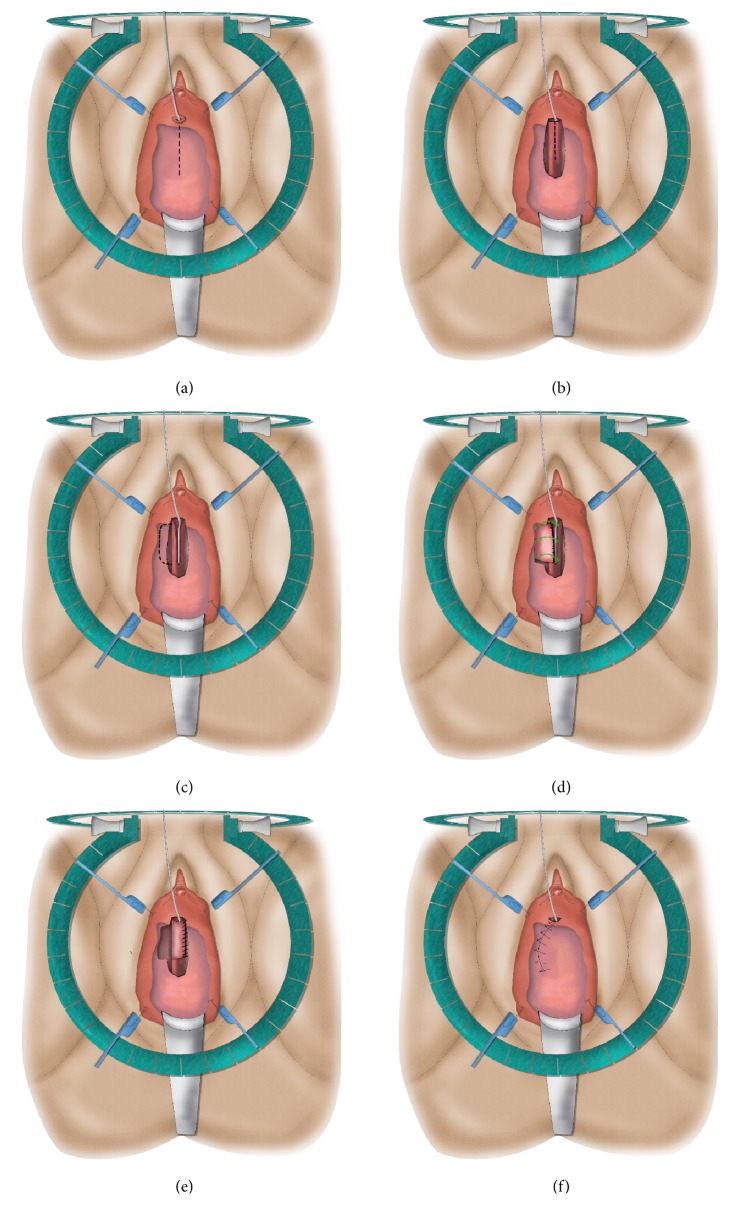
Lateral vaginal wall flap (“Orandi flap”).* (a) = longitudinal midline incision at anterior vaginal wall; (b) = ventral stricturotomy; (c) = mobilization of the lateral vaginal wall flap; (d) = suturing the medial surface of the flap towards the ipsilateral side of the urethra; (e) = turning the mucosal surface of the flap towards the urethral lumen and suturing the lateral side of the flap to the contralateral side of the urethra.*

*Lateral Vaginal Wall Flap (“Orandi Flap”) (Figure 7)*. This technique is inspired by the ventral longitudinal island penile skin flap in male urethroplasty and hypospadias reconstruction [14, 16]. A midline [14] or slightly lateral C-shaped [16] incision is made at the anterior vaginal wall. Dissection is directed towards the ventral urethra and the urethra is opened at the level of the stricture. The stricture is further opened along the guidewire until healthy proximal urethra is identified. A 2 cm wide flap with a length according to the length of the opened urethra is harvested from the lateral vaginal wall. Medially, the dissection of the flap is performed deep along the periurethral tissues. Laterally, the dissection is done along a superficial submucosal plane. This creates a laterally based vascular pedicle. The mobilization of this pedicle must be done sufficiently in order to allow the mucosal flap to be turned and sutured into the opened urethra. If hemostasis is needed, meticulous bipolar hemostasis is advised in order not to damage the vascularization of the flap. The medial surface of the flap is sutured towards the ipsilateral side of the urethra. The mucosal surface of the flap is turned towards the urethral lumen and the initial lateral side of the flap is sutured to the contralateral side of the urethra. The vaginal wall is closed with interrupted Donati sutures 2.0 above this reconstruction.

#### 4.2.3. Free Graft Urethroplasty

A multitude of grafts (vaginal, labial, buccal, or lingual mucosa) have been described in female urethroplasty [4]. In order to promote imbibition and inosculation, grafts require suturing onto a well-vascularized graft bed and the graft itself needs to be carefully defatted.

**Figure 8:**
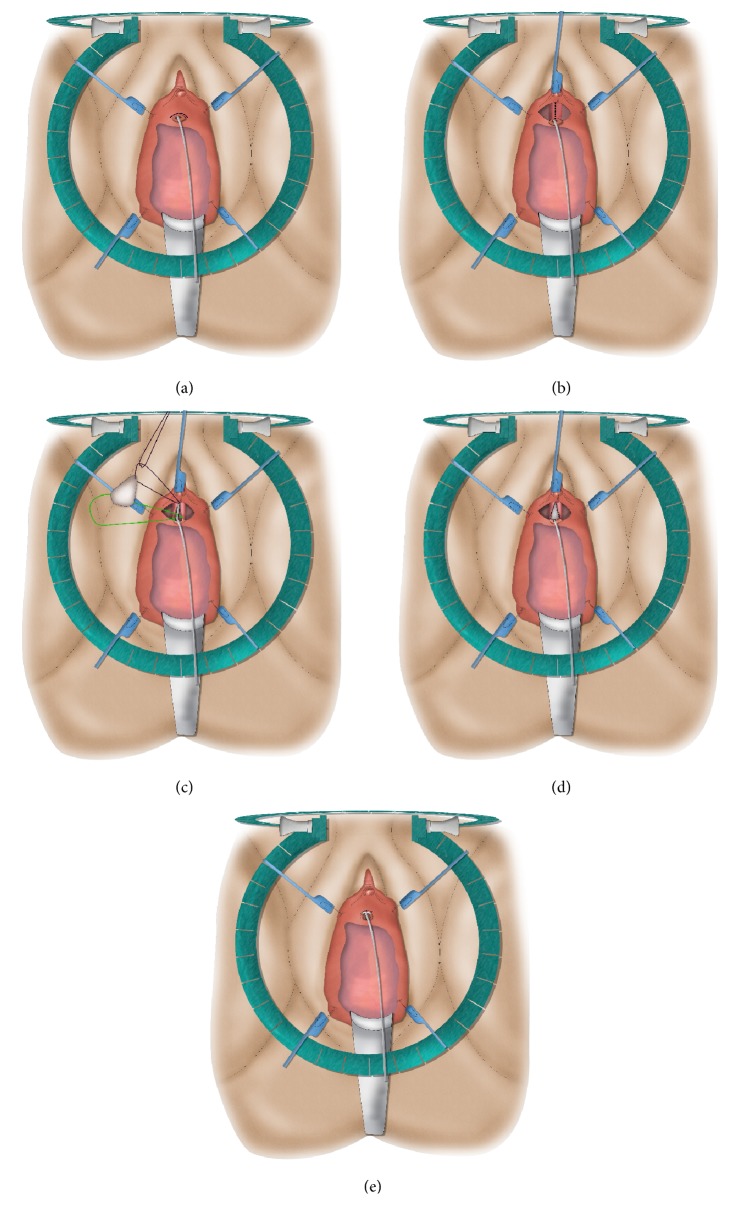
Dorsal onlay free graft urethroplasty.* (a) = semilunar suprameatal incision; (b) = dorsal stricturotomy; (c) & (d) = suturing the edges of the graft to the urethral edges; (e) = suturing the distal edges of the graft to the edge of the suprameatal incision.*

**Figure 9:**
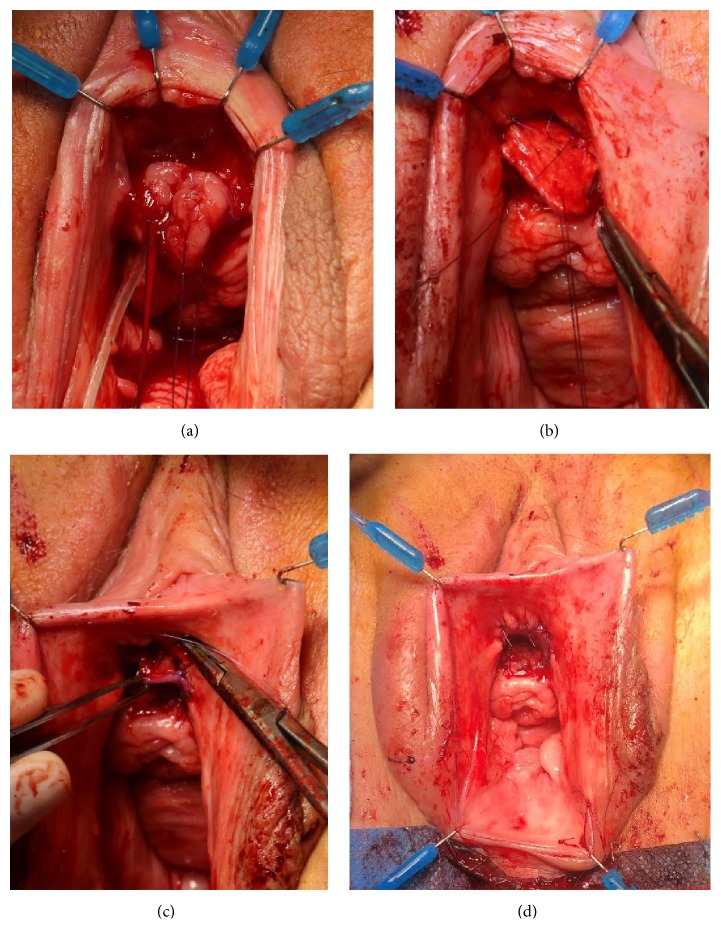
Dorsal onlay buccal mucosa graft urethroplasty.* (a) = semilunar dorsal incision and stricturotomy with stay sutures placed at the opened urethra; (b) = quilting the graft against the surface of the clitoral bodies and suturing the edges of the graft against the urethral edges; (c) = suturing the distal edges of the graft to the edge of the suprameatal incision; (d) = final result.*

*Dorsal Onlay (Figures 8 and 9)*. A semilunar suprameatal incision is made. The plane between the clitoris bodies and the dorsal urethra is dissected. The pubic bone is digitally palpated and marks the point of proximal dissection [18]. The dorsal urethra is incised and the stricture is opened along the guidewire until healthy proximal urethra is encountered. Stay sutures are placed at the urethral edges immediately after opening of the stricture. A graft is harvested according to the dimensions of the stricture. The graft is placed with its mucosal surface towards the urethral lumen. The edges of the graft are sutured to the edges of the opened urethra with a bilateral running suture. Suturing is started at the proximal urethra and continued up to the meatus. This suturing must also include the periurethral tissues in order to have a good fixation of the graft to the surrounding tissues. In addition to this, the graft is quilted to the clitoral bodies at the midline with resorbable sutures [8, 18]. At the meatus, the edges of the distal graft are approximated to the edge of the suprameatal incision with separate simple sutures [8].

**Figure 10:**
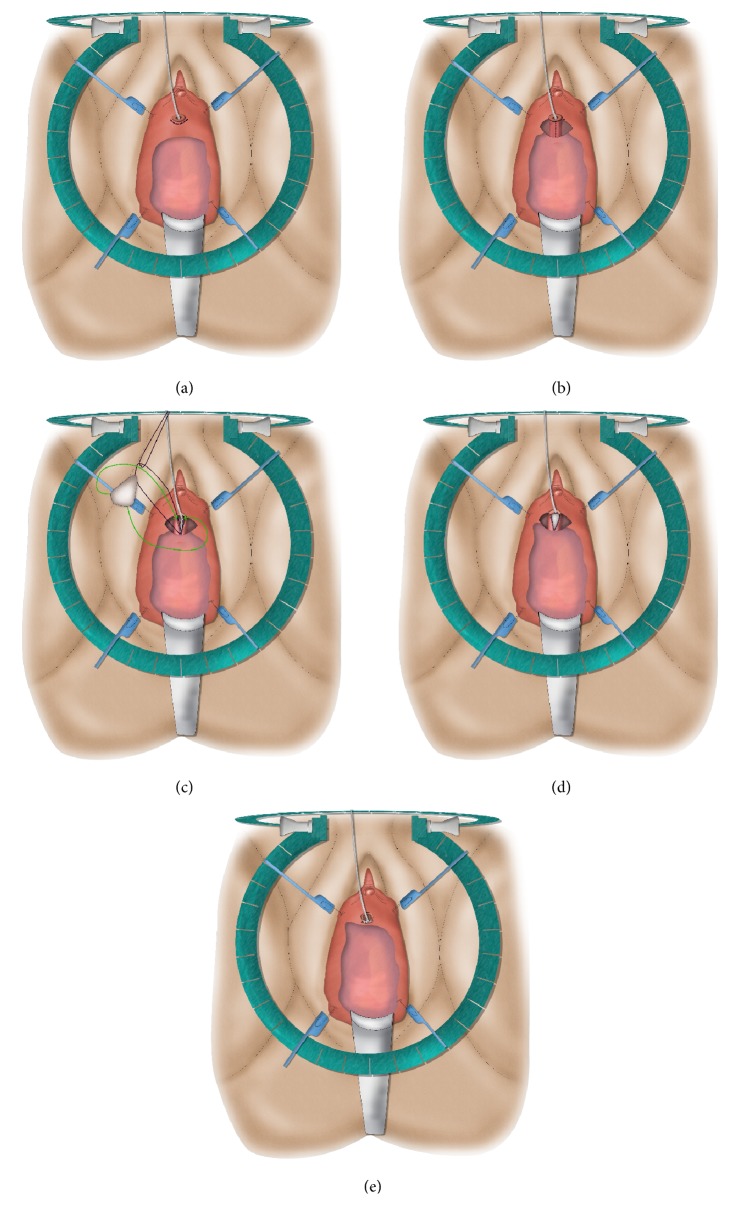
Ventral onlay free graft urethroplasty.* (a) = semilunar inframeatal incision; (b) = ventral stricturotomy; (c and d) = suturing the edges of the graft to the urethral edges; (e) = suturing the distal edges of the graft to the edge of the inframeatal incision.*

**Figure 11:**
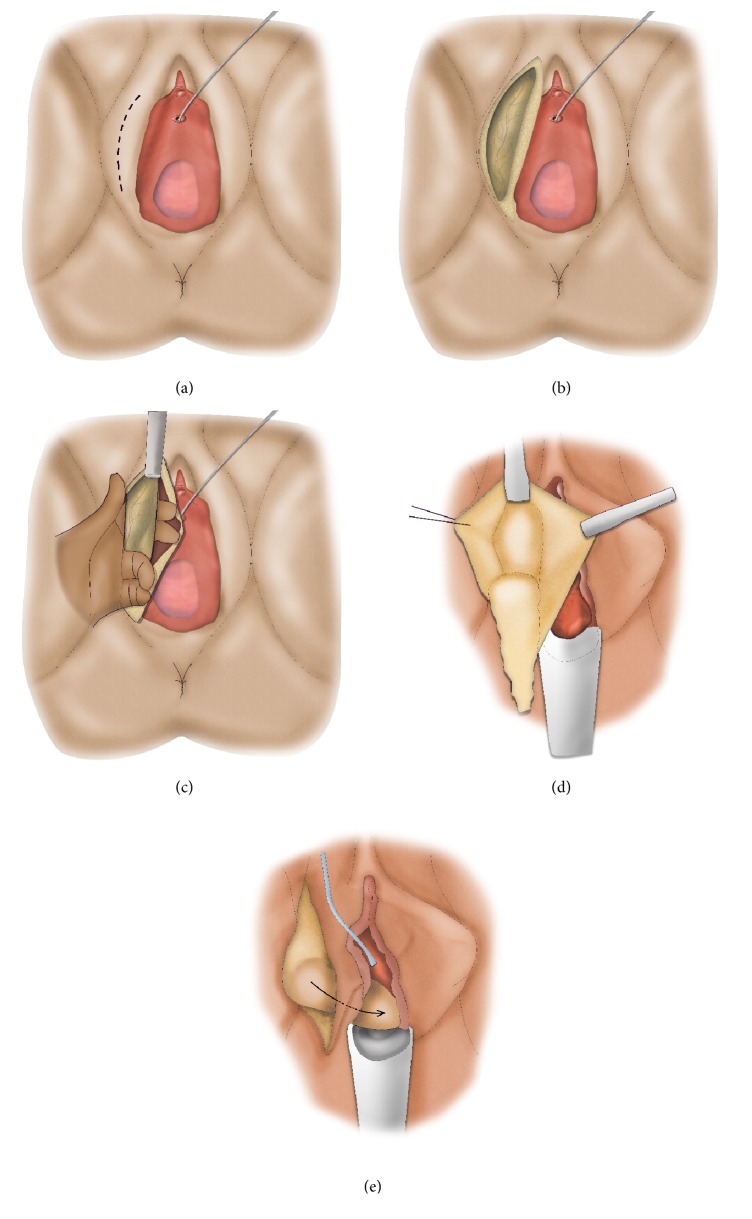
Martius flap.* (a) & (b) = sagittal incision at the most dependent line of the labium majus; (c) & (d) = mobilizing fibrofatty tissue; (e) = transposition of the Martius flap to the reconstructed area through a subcutaneous tunnel.*

**Figure 12:**
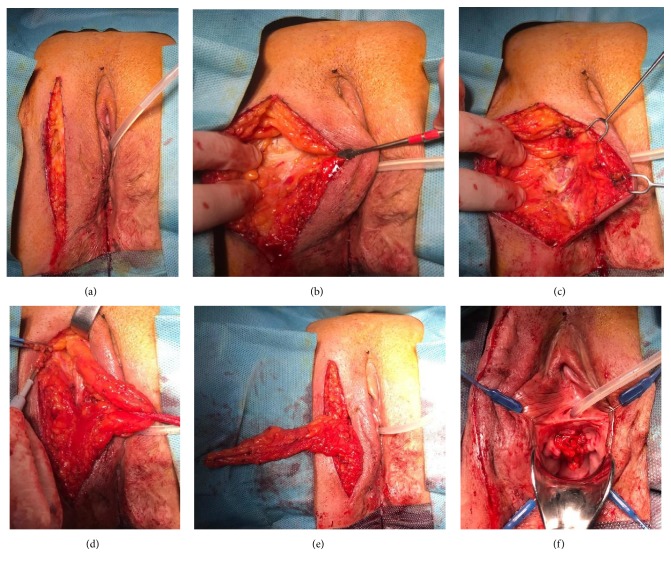
Martius flap procedure after ventral urethral repair.* (a) = sagittal incision line; (b) = lateral dissection to the labiocrural fold; (c) = medial dissection up to the bulbospongiosus muscle; (d) = mobilization of the flap; (e) = division of the anterior pedicle; (f) = transposed Martius flap to the ventrally reconstructed area.*

*Ventral Onlay (Figure 10)*. Development of the plane between the vaginal wall and the urethra can be facilitated by hydrodissection using a diluted solution of lidocaine with epinephrine [10]. A midline incision in the anterior vaginal wall is made above the region of the stricture. Dissection is performed towards the urethra and semilunar from the 3-o'clock to the 9-o' clock position around the ventral urethra. A ventral midline stricturotomy is performed at the level of the stricture. This stricturotomy can be started distally at the tip of a catheter or metal sound inserted through the meatus. Alternatively, stricturotomy can be started at the proximal end of the stricture. This is identified by the inflated balloon of a Fogarty embolectomy catheter that was inserted in the bladder and retracted up to the proximal end of the stricture [10]. The proximal and distal end of the stricture must allow passage of a 20Fr catheter after spatulation. Stay sutures are placed at the urethral edges to facilitate exposure. A graft is harvested according to the dimensions needed to augment the strictured urethra. The surrounding spongiosal tissue is sutured above this graft to provide a vascular bed. In case of insufficiency or poor quality of the vascular bed, a Martius flap must be mobilized towards the urethral reconstruction in order to provide an additional healthy vascular bed for the graft, to prevent urethra-vaginal fistula formation, and to ensure a healthy layer if subsequent suburethral sling insertion might be necessary (Figures 11 and 12) [10].

To harvest the Martius flap [19], a sagittal incision is made at the most dependent line of the labium majus. Dissection is proceeded until the deep fibrofatty tissue (“bright yellow”) is identified. This fibrofatty pad is laterally and medially mobilized following a natural tissue plane under the subcutaneous fat. Laterally, the flap is mobilized until the labiocrural fold. Medially, one should take care not to dissect into the bulbospongiosus and ischiocavernosus muscles. The flap is provided by a branch of the internal pudendal artery coming from posteriorly and a branch of the external pudendal artery coming from anteriorly. In most cases, the flap is pedicled at its posterior branch and the anterior pedicle is ligated with mobilization of the anterior part. The deep aspect of dissection takes place alongside the surface of the pubic bone [20]. A subcutaneous tunnel (2 fingers wide) is created between the vaginal and the labial incision and the flap is transposed to the site of urethral reconstruction [19]. The flap is quilted to the graft with interrupted absorbable sutures 5.0. Abundant tissue of the flap is resected. A suction drain is left in place at the labial incision and the wound is closed in layers above this [19]. The vaginal epithelium is closed over the flap with 3.0 absorbable sutures (running suture or Donati) (Figures 11 and 12).

## 5. Postoperative Care

Vaginal packing during 24 h is advised for hemostatic reasons [10, 18].

At the end of the procedure, a 16 to 20 Fr urethral catheter is inserted through the reconstructed urethra. In case of a vestibular flap, the catheter can be removed early, even after 1 day [9]. After an anterior vaginal wall flap, the catheter is maintained for 7-10 days [17]. After a lateral vaginal wall flap, the catheter is maintained for 3 weeks [14, 16]. For ventral and dorsal onlay graft urethroplasty, the catheter remains for 2-3 weeks [8, 10, 18]. The catheter is removed if there is no contrast extravasation on urethrography.

## 6. Choice of Technique

At present, there are no guidelines on how to treat female urethral strictures despite the multitude of techniques that have been published [18]. In general, a trial of internal urethrotomy and/or dilation is performed initially [3]. Based on a systematic review of Osman et al., the composite success rate is 47% with a 58% and 27% success rate in case of, respectively, no previous dilations or previous dilations [4]. As most of these women were performing intermittent catheterization afterwards, this success rate is too optimistic. This practice must be considered as a form of repetitive dilations and should be discouraged in symptomatic women. All types of urethroplasty are associated with a higher success rate. Vaginal flap urethroplasty, vaginal/labial graft urethroplasty, and oral mucosa graft urethroplasty have a composite success rate of, respectively, 91%, 80%, and 94% [4]. No large and well-conducted comparative trials have been performed to evaluate whether one technique is superior to another, whether the dorsal location is better than the ventral one or whether a specific type of graft performs better than the others. The choice of technique therefore is mainly dictated by the treating surgeon's experience and preference. Nevertheless, based on the general principles of surgery and wound healing, based on the experiences in male urethroplasty and based on expert opinion, some advices can be suggested:

- *Heineke-Mikulicz meatoplasty*: Despite the high success rate (96%) [11], this technique can only be used for very short (<0,5 cm) meatal strictures [3]. When applied for longer strictures, it will result in a hypospadiac meatus with vaginal voiding and irritation. Furthermore, this technique is not advised in case of lichen sclerosus as this disease will further affect the reconstructed meatus [21].
- *Use of genital mucosa (vaginal/labial)*: In male urethroplasty, the use of genital skin in case of lichen sclerosus resulted in an up to 100% failure rate [21]. Based on this observation, genital mucosa should be avoided in females with lichen sclerosus as stricture etiology. Instead, the use of oral mucosa is advised as it is more resistant to lichen sclerosus. In case of vaginal atrophy, vaginal mucosa (graft or flap) is not suitable for urethral reconstruction [3, 4, 8, 10]. Even in women with normal genital mucosae, atrophic changes will occur after the menopause. This might affect the reconstruction as well and can be a cause of future stricture recurrence [10]. Long-term follow-up after urethroplasty with genital mucosa is needed to accept/reject this hypothesis. Vaginal mucosa graft is also not advised in case of a narrow vaginal introitus as this will further exacerbate this [8]. No matter what type of graft is used, it must be sutured onto a well-vascularized graft bed to ensure graft survival and success of the urethral reconstruction. In addition, the graft must be immobilized at the graft bed as much as possible. Quilting sutures to the graft bed are important for this purpose.
- *Anterior vaginal wall and vestibular flaps* can be used for meatal stricture and short (<2cm) distal urethral strictures [14]. The anterior vaginal wall flap can cause an inward urine stream with vaginal voiding [14, 22]. The vestibular flap has the potential disadvantage of spraying and anterior deflection of the urinary stream. Longer strictures (>2cm) or proximal stricture must be treated with graft procedures or lateral vaginal flap urethroplasty [10, 16].
- *Ventral procedures* are technically more easy to perform [4]. Furthermore, due to the omega-shape of the female urethral sphincter with its ventral midline deficiency, ventral stricturotomy and subsequent urethroplasty should have less risk of stress urinary incontinence [3, 10]. However, this hypothesis seems to be solely theoretical as no excess in stress urinary incontinence has been reported with dorsal procedures as well [4]. The paucity of ventral muscular tissue and overlapping suture lines after reconstruction pose a risk for the development of urethrovaginal fistula formation [3, 4]. In ventral free graft procedures, a low burden for the use of the Martius flap should be maintained especially in case of poor quality of the ventral local tissues. In addition, the use of a Martius flap makes subsequent insertion of a suburethral sling possible [4]. Its use should be balanced against the complications of the Martius flap which are in general minor (labial hematoma, cosmetic labial problems, decreased sensitivity, and local pain) [19, 20].
- *Dorsal procedures* are technically more challenging with more risk of bleeding and damage to the clitoral bodies [3]. The fear of injury to the clitoral neurovascular bundle seems not to be justified [4]. On the other hand, the dorsal approach has less risk of fistula formation and graft sacculation and is preferred if future insertion of a suburethral sling is expected and in case of fibrosis or unhealthy appearance of the ventral vaginal wall [2, 4, 8, 15, 18].

## 7. Conclusions

Female urethroplasty provides excellent cure rates and must be performed in case of recurrence after dilation. Before urethroplasty, diagnostic modalities are needed to confirm the presence, the location, and length of the stricture and to provide insight into the quality of surrounding tissues. The choice of technique depends on stricture length, stricture location, and the quality of local tissues. Nevertheless, the optimal treatment strategy in female urethral strictures needs further clarification, preferably with larger and comparative series.
